# Cyclin D1 and CHEK2 polymorphic variants as low risk susceptibility alleles in DTC patients

**DOI:** 10.1186/1897-4287-13-S1-A15

**Published:** 2015-09-09

**Authors:** Szymon Hryhorowicz, Katarzyna Ziemnicka, Marta Kaczmarek-Rys, Justyna Hoppe-Golebiewska, Andrzej Plawski, Marzena Skrzypczak-Zielinska, Monika Golab, Malgorzata Szkudlarek, Batlomiej Budny, Marek Ruchala, Ryszard Slomsk

**Affiliations:** 1 The NanoBioMedical Center, Adam Mickiewicz University, Poznan, Poland; 2 The Department of Endocrinology, Metabolism and Internal Diseases, Poznan University of Medical Sciences, Poznan, Poland; 3 Institute of Human Genetics, Polish Academy of Sciences, Poznan, Poland; 4 Department of Biochemistry and Biotechnology, University of Life Sciences, Poznan, Poland

## 

Differentiated thyroid cancer (DTC) originates from thyroid follicular epithelial cells and belongs to a group of slowly progressing tumors with relatively good prognosis. However, optimal results in the treatment of these tumors via coordinated multimodal therapy are achieved mostly by early diagnosis. A serious problem in course of DTC are recurrences and metastasis. Conversion from well differentiated thyroid cancer to aggressive anaplastic carcinoma is possible, which underlines the importance of effective diagnosis and characterisation at the molecular level as well. A genetic background of DTC is not clear. There are known several mutations detected in the thyroid tumor cells, but genetic factors predisposing to promotion of carcinogenesis of the thyroid gland are still unexplained.

The *CHEK2* gene (checkpoint kinase 2) is located in position 22q12.1 and spans 54.6 kb. It encodes the human ortholog of the yeast checkpoint kinases Cds1 and Rad53. In response to the damage of genetic material and inhibition of the replication process, the protein is activated by phosphorylation. Mutations in the *CHEK2* gene are evidenced to be a multicancer predisposition factor. The protein product of this gene is a cell cycle checkpoint regulator, tumor suppressor and is a member of subfamily of serine/threonine protein kinases.

The *CCND1* gene (11q13) encodes the cyclin D1, a protein which belongs to the highly conserved cyclin family, that serve as regulators of CDK kinases. Cyclin D1 is a key regulatory protein which plays an important role in the transition from the G1 to the S phase of cell cycle during cell division. The cancer relevance of the cyclin D1 gene was apparent upon its identification in 1991. The cyclin D1 locus was initially identified based on its involvement in a chromosomal rearrangement of benign parathyroid tumors. The *CCND1* gene alterations which influence cell cycle progression are frequently observed in a variety of tumors and may contribute to tumorigenesis.

The aim of the study was to examine two sequence variants in *CHEK2* p.R145W (c.433C>T, rs137853007 and p.I157T c.470T>C, rs17879961)and *CCND1 (*c.723G>A (rs9344; p.Pro241= and c.669C>T, rs3862792; p.Phe223=) genes in a group of Polish patients with differentiated thyroid cancer and control individuals from Polish population. We examined a cohort of 647 Polish patients diagnosed with differentiated thyroid carcinoma and Polish population included 799 subjects (615 females and 184 males).

As a genotyping technique we used pyrosequencing. In order to determine statistical significance of differences in genotypic and allelic frequency, observed among compared groups, we used GraphPad Prism 4 (chi-square distribution and Fisher exact test).

We did not observe noteworthy variability in p.R145W (c.433C>T, rs137853007), but in analysis of p.I157T sequence variant (c.470T>C, rs17879961) we demonstrated statistically significant differences in allele frequencies (p=0.005, OR=2.014, C.I.=[1.348-3.010]).

In a case of *CCND1* gene we observed statistically higher frequency of allele A in rs9344 locus in DTC patients (p=0.02498, OR=1.191, C.I.=[1.022-1.387]). We observed also a statistically significant difference between genotypes in subjected group. Genotype AA occurs significantly to be more frequent in patients with DTC (p=0.02023, OR=1.452, C.I.=[1.059-1.989]) than in control population group. We did not notice any statistically important differences in frequencies of genotypes or alleles in rs3862792 locus in subjected groups.

In conclusion the allele c.470C *CHEK2* gene and c.723A variant *CCND1* gene may be a risk alleles in the development of differentiated thyroid cancer in Polish population.

